# IN SEARCH OF TIME: TIME PERSPECTIVE AND LIFE’S FINITUDE

**DOI:** 10.1093/geroni/igae098.0369

**Published:** 2024-12-31

**Authors:** Yaeji Kim-Knauss, Clara Paula de Couto, David Ekerdt

**Affiliations:** Chair; Co-Chair; Discussant

## Abstract

Human existence is inherently bounded by a finite timeline, and this reality shapes our perceptions, attitudes, and behaviors towards the time. This awareness of life’s finitude prompts individuals to prioritize meaningful pursuits or to adapt to the constraints of time, as elucidated in theories in gerontology, and validated through numerous empirical studies. Against this background, this symposium delves into the impact of time perspectives on psychological outcomes, as well as on individuals’ approaches to their own mortality. N. Fung and H. Fung will offer a comprehensive examination of how motivation changes as time left in life is perceived to decrease. Wirth et al. will present findings about the influences of different subsets of time perspective on life satisfaction using longitudinal data. Incorporating ideas from construal-level theory, de Paula Couto et al. will delve into age-specific factors associated with desired longevity under impairment conditions. Kim-Knauss et al. will discuss behaviors associated with perceived engagement in end-of-life planning and examine potential cultural differences by comparing the findings from Germany and South Korea. Selovin et al. will explore psychological and behavioral factors contributing to a sense of preparedness for death and dying among older adults. Finally, Ekerdt will discuss the findings from these presentations, offering comprehensive insights into the interplay between time perspective and human behavior in confronting mortality.

